# Cardiac dyssynchrony in a Becker cohort: preliminary results

**DOI:** 10.1186/1532-429X-17-S1-P363

**Published:** 2015-02-03

**Authors:** Raymond Gilles, Karim Wahbi, Marcel Toussaint, Benjamin Marty, Pierre Carlier

**Affiliations:** 1 Cardiology, CHWAPI Tournai, Tournai, Belgium; 2 NMR laboratory, Institut de Myologie - Pitié-Salpétrière, Paris, France; 3 Institut de Myologie - Pitié-Salpétrière, Paris, France

## Background

We previously reported the early detection of cardiac motion dyssynchrony in GRMD dogs (a model for Duchenne myopathy with cardiac involvement). In this new work, we applied the same dyssynchrony index in a cohort of 88 Becker patients of various age (38,7 ±13,6 years; range from 18 to 68) and disease burden (number of segment count with any Gd late enhancement : 4,37 ± 3,14 ; range from 0 to 12). We compared the data obtained to those in 10 control subjects.

## Methods

Images were acquired on a Siemens Magnetom Trio 3T® and analyzed using Segment® software (Medviso AB). For each segment (AHA classification) of the two most basal short axis slices studied on MR cine true-FISP images, we assess the standard deviation of the mean position of the inner heart border. The mean for all phases of the cardiac cycle and both slices equals the global dyssynchrony index (DI). At this point, we do not dissociate systolic and diastolic dyssynchrony.

## Results

For our 88 Becker patients, the DI is 6,99 ± 3,16 while it is 5,17 ± 1,07 in the controls (p <0,001). There is some correlation between DI and LVEF (r = 0,55 ; p<0,05). Even if the higher DIs can be seen in some severely impaired hearts, the high DIs however exist all through the patient sample (see figure).

**Figure 1:**
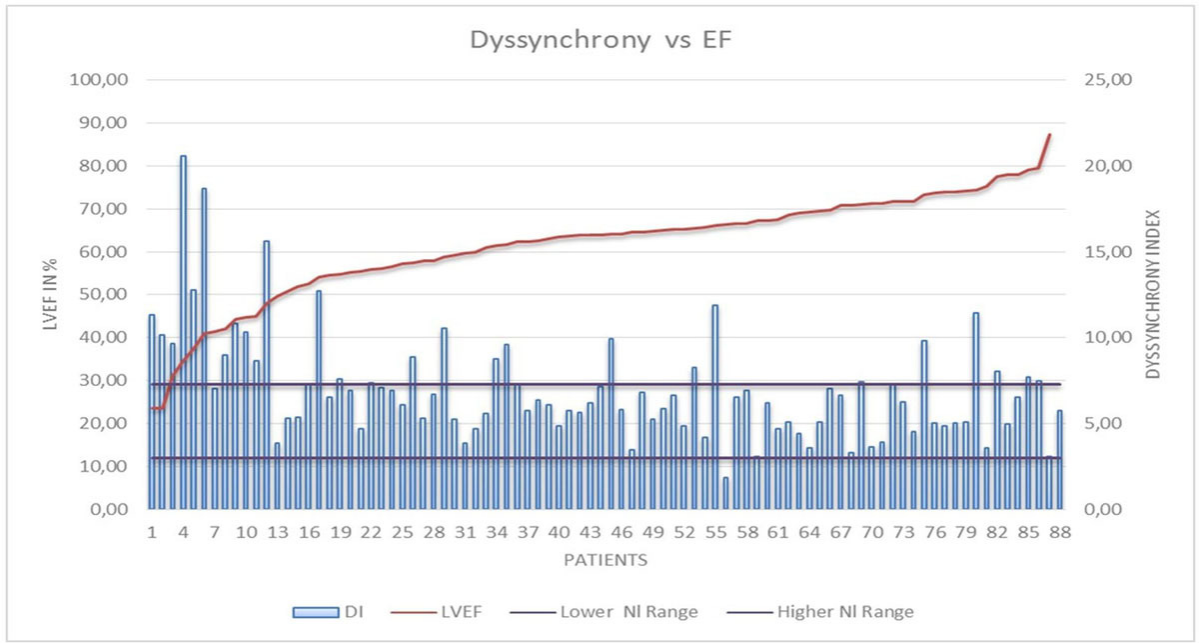
For each individual patient in the cohort, from the lowest LVEF on the left to the highest on the right, the dyssynchrony index (DI) measured is displayed and compared to the normal range of DI values in the control subjects.

We found no correlation between the dyssynchrony index and the presence of Gd late enhancement in all segments (r=0,24) or in the lateral segments (r=0,07). We investigated the lateral segments more specifically because it is known from previous studies that the lateral wall is an early target of Gd late enhancement in Becker's disease.

## Conclusions

Dyssynchrony in cardiac motion is globally higher in Becker patients compared to controls. Although part of the anomaly is probably driven by systolic asynchrony in very low EF patients, some patients with normal EF show dyssynchrony as well. We found no correlation between the dyssynchrony index and the presence of Gd late enhancement.

## Funding

N/A.

